# Hemodialysis Increases the Risk of Lower Gastrointestinal Bleeding and Angiodysplasia Bleeding: A Nationwide Population Study

**DOI:** 10.1155/2020/7206171

**Published:** 2020-03-03

**Authors:** Tzung-Jiun Tsai, Wen-Chi Chen, Yu-Tung Huang, Yi-Hsin Yang, I-Che Feng, Wen-Chieh Wu, Huang-Ming Hu, Deng-Chyang Wu, Ping-I Hsu

**Affiliations:** ^1^Division of Gastroenterology and Hepatology, Department of Internal Medicine, Kaohsiung Veterans General Hospital, Kaohsiung, Taiwan; ^2^National Yang-Ming University, Taipei, Taiwan; ^3^Center for Big Data Analytics and Statistics, Chang Gung Memorial Hospital, Linkou, Taiyuan, Taiwan; ^4^Center for Medical Informatics and Statistics, Kaohsiung Medical University, Kaohsiung, Taiwan; ^5^Division of Gastroenterology and Hepatology, Department of Internal Medicine, Chi-Mei Medical Center, Tainan, Taiwan; ^6^Division of GERD Center, Yuan Sheng Hospital, Changhua, Taiwan; ^7^Division of Gastroenterology & Hepatology, Department of Internal Medicine, Kaohsiung Medical University Hospital, Kaohsiung, Taiwan; ^8^Department of Medicine, An Nan Hospital, China Medical University, Taiwan

## Abstract

**Background:**

Patients with chronic kidney disease (CKD) with or without hemodialysis were considered to have bleeding tendency and higher risk for gastrointestinal (GI) bleeding. Previous studies had documented that hemodialysis may increase the gastroduodenal ulcer bleeding. Few studies evaluated the relationship between CKD and lower GI bleeding. *Materials and Methods*. An observational cohort study design was conducted. The end-stage renal disease (ESRD) patients receiving regular hemodialysis (dialysis CKD), CKD patients without dialysis (dialysis-free CKD), and controls were selected from 1 million randomly sampled subjects in the National Health Insurance Research Database of Taiwan. These three group subjects were matched by age, sex, comorbidity, and enrollment time in a 1 : 2 : 2 ratio. The Cox proportional hazard regression models were used to identify the potential risk factors for lower gastrointestinal bleeding.

**Results:**

Dialysis CKD patients (*n* = 574) had a higher incidence of lower GI bleeding than dialysis-free CKD patients (*n* = 574) had a higher incidence of lower GI bleeding than dialysis-free CKD patients (*n* = 574) had a higher incidence of lower GI bleeding than dialysis-free CKD patients (*P* < 0.001). Multivariate analysis showed that extreme old age (age ≥ 85), male gender, dialysis-free CKD, and dialysis CKD were independent factors of lower GI bleeding. Additionally, dialysis CKD patients also had a higher incidence of angiodysplasia bleeding compared to dialysis-free CKD patients and control subjects (1.1% vs. 0.1% and 0.1%, respectively; both *P* < 0.001). Multivariate analysis showed that extreme old age (age ≥ 85), male gender, dialysis-free CKD, and dialysis CKD were independent factors of lower GI bleeding. Additionally, dialysis CKD patients also had a higher incidence of angiodysplasia bleeding compared to dialysis-free CKD patients and control subjects (1.1% vs. 0.1% and 0.1%, respectively; both

**Conclusion:**

Hemodialysis may have higher risk of lower GI bleeding and angiodysplasia bleeding.

## 1. Introduction

End-stage renal disease (ESRD) under regular renal replacement treatment, such as hemodialysis (HD), peritoneal dialysis, and transplantation, is a worldwide public health problem. The global prevalence of ESRD is 280 per million population [1]. According to the most recent United States Renal Data System (USRDS) database, the prevalence of CKD was 15%, and ESRD was 2160.7 per million in the United States populations [2]. The prevalence of chronic kidney disease (CKD) in Taiwan was 9.8-11.9% [3–6]. According to the most recent data, the prevalence of CKD in Taiwan increased from 11.9% in 2010 to 15.46% in 2018 [3, 7], and the incidence increase from 13.5/1000 person-years in 2003 to 27.21/1000 person-years in 2018 [7, 8]. Taiwan had higher prevalence and incidence rate of CKD, which might be due to the aging country and low public perception of the renal disease. A recent meta-analysis showed that ESRD increased the mortality, need for transfusion, rebleeding rate, and length of hospitalization in gastrointestinal (GI) bleeding patients [9]. Several cohort studies also documented that patients with CKD had higher incidences of upper GI bleeding [10, 11], peptic ulcer bleeding [12–14], and recurrent upper GI rebleeding [15–17] than those without CKD [18]. However, few studies investigate the exact incidence of lower GI bleeding and angiodysplasia bleeding in patients with CKD with or without hemodialysis. Most cohort studies for lower GI bleeding were from the West. Few Eastern countries' studies were seen.

Lower GI bleeding is an emerging problem for medical doctors. A population-based epidemiological study from Spain demonstrated that the lower GI event rate was increasing from 1996 to 2005 though the upper GI bleeding, and perforation rates were decreasing [19]. The study also showed that lower GI events displayed a higher mortality rate, longer hospitalization, and higher resource utilization than upper GI bleeding [19]. In addition, another study by Patel et al. reported that lower GI bleeding increased mortality rate of in patients with coronary artery disease on triple antithrombotic therapy [20].

Angiodysplasia is an important cause of lower GI bleeding [21]. It is more common in patients with chronic renal failure, and its prevalence is related to the severity of the renal disease [22]. Angiodysplasia bleeding accounts for 19-32% of lower GI bleeding episodes in patients with chronic renal failure compared with 5-6% of bleeding episodes in general population [23, 24].

The aims of this study were to investigate the incidence of the lower GI bleeding in dialysis CKD and dialysis-free CKD patients and to identify the factors that are predisposing to the lower GI bleeding.

## 2. Materials and Methods

An observational cohort study design was conducted. The CKD patients receiving regular hemodialysis (dialysis CKD), CKD patients without hemodialysis (dialysis-free CKD), and controls were selected from 1 million randomly sampled subjects in the National Health Insurance Research Database (NHIRD) of Taiwan. The National Health Insurance (NHI) is the universal health insurance coverage more than 99% residents in Taiwan.

The study protocol was approved by the Institutional Review Board in Kaohsiung Veterans General Hospital, with the number 17-CT8-04 (170627-1).

### 2.1. Study and Control Group

The more than 20-year-old dialysis CKD patients were selected from the NHIRD as the study group between 2000/1/1 and 2012/12/31. And then using the dialysis CKD data in 1 : 2 : 2 to matched by age, gender, comorbidity, and enrollment time with dialysis-free CKD and controls from the same dataset and time periods.

The included subjects were extracted based on the International Classification of Disease, 9th revision, Clinical Modification (ICD-9-CM). Dialysis CKD patients were identified by ICD-9-CM code 585 and with a catastrophic illness card. The index date of the dialysis CKD group was the first appearance of catastrophic illness card. Patients who received the catastrophic card had chronic renal failure and had been receiving regular hemodialysis for at least 3 months. Subjects of dialysis-free CKD were identified by ICD-9-CM 582.0, 582.4, 582.8x, 586, 250.4x, 274.1, 403.x1, 404.x2, and 404.x3, which occurred in the hospitalization claims, or when their names were encountered in the outpatient department 3 times continuously when documents were examined [12]. This definition may correspond to CKD stages 4 and 5. The index date of this group was defined as the first-time diagnosis of CKD.

We excluded patients with renal transplantation, peritoneal dialysis, cirrhosis, GI tract malignancy, inflammatory bowel disease, coagulopathy, vascular insufficiency of the intestine, radiation gastroenteritis or colitis, a history of gastrointestinal bleeding one year before the index date, and a medication history of gastroprotective agents (PPI and H2B) and ulcerogenic agents (NSAID), which had been used for at least 4 weeks in the 8 weeks before the index date.

### 2.2. End Point

The primary endpoint was lower GI bleeding, which was identified by patients who with any admission diagnosis of ICD-9-CM 562.02, 562.03, 562.12, 562.13, 569.3, 578.1, 578.9, and 569.86, and angiodysplasia bleeding with ICD-9-CM 537.83 and 569.85. The bleeding-related mortality was defined as the last admission with a discharge diagnosis of GI bleeding and discharge due to death or in critical status, or patient's withdrawal from the NHI. The incidences of all GI bleeding, lower gastrointestinal (LGI) bleeding, and angiodysplasia bleeding in patients with dialysis CKD, dialysis-free CKD, and the control groups were compared.

### 2.3. Statistical Analysis

The data were represented by frequency (percentage), and continuous variables were expressed as mean ± standard deviation. We compared continuous variables by Student's *t* test and/or analysis of variance (ANOVA). Categorical variables were tested by chi-squared test or Fisher's exact test. The time survival curve was evaluated by the Kaplan-Meier method to evaluate the cumulative incidence of lower GI bleeding. We compared the competing factors by log-rank test. Finally, we performed Cox proportional hazard regression analysis to evaluate the risk factor of lower GI bleeding. A two-sided *P* value of <0.05 was considered statistically significant.

## 3. Results

### 3.1. Demographic Data

**Figure 1:**
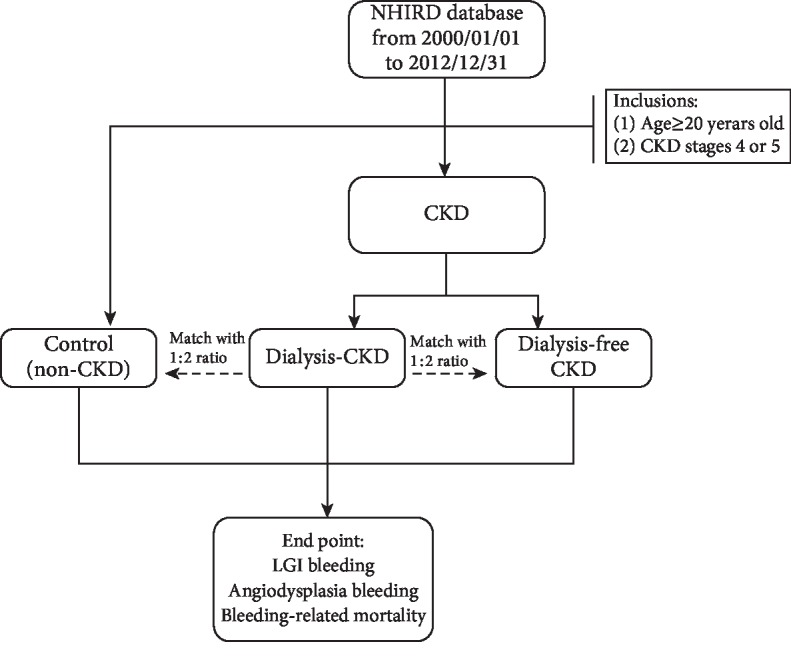
Flow chart of selection of patients with chronic kidney disease and matched control from the National Health Insurance Research Database of Taiwan.

A total of 574 dialysis CKD patients, 1148 dialysis-free CKD patients, and 1148 controls were selected from the one million NHIRD subjects (Figure 1). The demographic data of the dialysis CKD, dialysis-free CKD, and control groups are summarized in Table 1. The three study groups had comparable age, gender, and comorbidity. However, the dialysis CKD and dialysis-free CKD groups had higher Charlson scores than that of the control group (1.7 ± 1.5 and 1.5 ± 1.4 vs. 1.4 ± 1.3; *P* < 0.001). There were also no differences in the frequencies of antithrombotic agent use among the three study groups (Table 1).

### 3.2. The Rate of GI Bleeding

**Figure 2:**
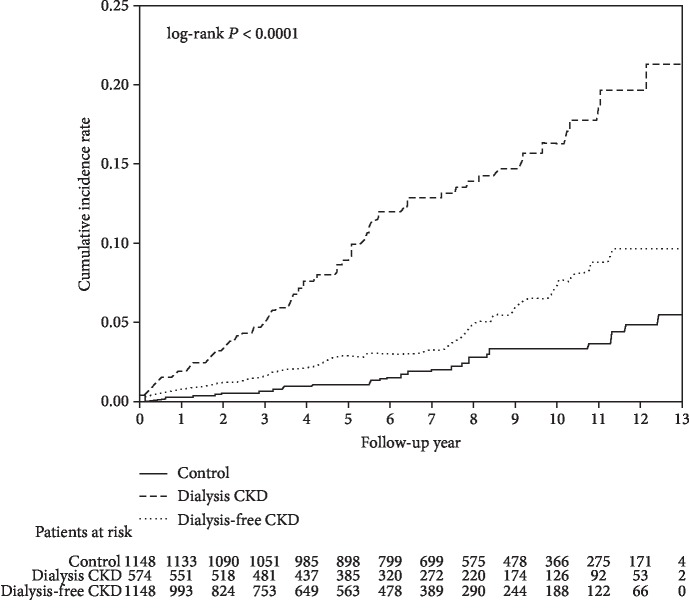
Cumulative incidence of lower gastrointestinal bleeding in dialysis CKD, dialysis-free CKD, and control groups.

During a mean follow-up of 6.4 years, a total of 175 patients (30.5%) in the dialysis CKD group suffered from GI bleeding episodes and needed hospitalization, with 126 (11.0%) in dialysis-free CKD, and 93 (8.1%) in the control group. Both dialysis CKD and dialysis-free CKD groups had higher incidences of GI bleeding than the control group (*P* < 0.001 and *P* = 0.019, respectively). In lower GI bleeding, dialysis CKD group also had higher rate compared to dialysis-free CKD and control groups (12.9% vs. 3.6% and 2.8%; both *P* < 0.001; Table 2). There were no differences in the incidence of lower GI bleeding between the dialysis-free CKD group and control group. Kaplan-Meier survival analysis showed that dialysis CKD group exhibited a higher incidence of lower GI bleeding than dialysis-free CKD and control groups (Figure 2; both *P* < 0.0001 by log-rank test). The dialysis CKD group had a higher incidence of angiodysplasia bleeding than dialysis-free CKD and control groups (1.1% vs. 0.1% and 0.1%; both *P* < 0.003, Table 2) although the case number was small. The dialysis-free CKD group and control group displayed comparable incidence of angiodysplasia bleeding (*P* = 1.00).

### 3.3. Risk Factors for Lower GI Bleeding

Table 3 lists the independent clinical factors influencing the incidence of lower GI bleeding. Multivariate analysis disclosed that extreme old age (age ≥ 85), male gender, dialysis CKD, and dialysis-free CKD were independent risk factors for lower GI bleeding with adjusted hazard ratios of 61.47 (95% confidence interval (CI): 2.68-1412.10), 3.14 (95% CI: 1.45-6.78), 29.09 (95% CI: 9.66-87.63), and 6.61 (95% CI: 2.27-19.23), respectively.

## 4. Discussion

In the nationwide population-based cohort study, we investigated the impact of CKD on the incidence of lower GI bleeding and assessed the incidences of angiodysplasia bleeding in patients with CKD and general population. The data clearly demonstrated that dialysis CKD patients had a higher incidence of lower gastrointestinal bleeding than dialysis-free CKD patients and control subjects (12.9% vs. 3.6% and 2.8%; both *P* < 0.001). Multivariate analysis documented that both CKD under hemodialysis and CKD without hemodialysis were independent factors predicting lower GI bleeding. Additionally, dialysis CKD patients also had a higher incidence of angiodysplasia bleeding than dialysis-free CKD patients and control subjects.

In the baseline characteristics, we found higher Charlson score in the dialysis CKD group. Charlson score included the parameter of severity of renal disease, and this might contribute to the result of the difference. Since Charlson score includes several diseases, and parts of the disease, such as diabetes, hypertension, and celebrovascular accidents, were included in the multivariate analysis, we did not put Charlson comorbidity index (CCI) into multivariate analysis. Further, putting CCI into the analysis would have collinearity or over-control problem.

A retrospective cohort study from Canada showed that the 3-year cumulative incidence of lower GI bleeding in ESRD patients receiving chronic dialysis was 8.9% [25]. In the current study, the annual incidences of lower GI bleeding in the dialysis CKD, dialysis-free CKD, and control groups were 0.80%, 0.32%, and 0.19% person-years, respectively. The dialysis CKD group had a higher cumulative incidence of lower GI bleeding than dialysis-free CKD and control groups (12.9% vs. 3.6% and 2.8%). Multivariate analysis revealed that ESRD was an independent risk factor for lower GI bleeding with an adjusted hazard ratio of 29.1 (95% CI: 9.7-87.6). The promising data presented here are consistent with an independent work in an USA community-based study [26] showing a higher incidence of lower GI bleeding in patients with eGFR (ml/min per 1.73 m^2^) < 30. The work revealed that the hazard ratios of lower GI bleeding in patients with eGFR≧90, 60-80, 30-59, and <30 were 1, 1.5, 2.3, and 10.8, respectively.

In the Cox regression analysis, Table 3, we had 15 control variables. According to rule of thumb, Cox model outcome events (at least 10 events per variable (EPV)), we need at least 150 events to get stable estimates of the regression coefficients. In our data, we found 147 lower GI bleeding events, and a little lower than the EPV recommendation. Further result interpretation should be cautious. But in our data, we reported the rare event rates in the real would.

Angiodysplasia is a common and important cause of GI bleeding, especially in patients with CKD. Angiodysplasia bleeding was reported to be associated with chronic renal failure, Von Willebrand's disease, aortic stenosis, cirrhosis, and pulmonary disease, but the actual etiology is unknown [23]. Currently, the pathophyiological mechanisms contributing the development of angiodysplasia and consequent bleeding in patients with chronic renal failure are unclear. Several possible causes including uremic platelet dysfunction [27, 28] and use of anticoagulants [29] have been proposed to explain the increased risk of bleeding in uremic patients. Further studies are warranted to investigate the pathogenesis of the development of angiodysplasia bleeding in patients with ESRD.

In addition to CKD and hemodialysis, extreme old age, and male gender were independent risk factors predicting lower GI bleeding. Previous studies reported that the prevalence of diverticular disease bleeding and angiodysplasia bleeding increases with age [30], which might explain why old age is associated with higher risks of LGI bleeding. The actual cause of males having a higher risk of lower GI bleeding is unclear. According to previous literature review, males are at higher risk of GI bleeding [19, 31]. Further study is needed to confirm the reason for this.

Anticoagulant and antiplatelet agents are reported to be the risk factors for lower GI bleeding [32, 33], but still some authors stated that antiplatelet agents were not associated with LGI bleeding [34]. In our study, we found that LGI bleeding had no association with antiplatelet agents and anticoagulants.

There were some limitations to this study. First, this was a retrospective cohort study, and immortal time bias and selection bias might be present in this study. Nonetheless, we have conducted multivariate analysis to identify the independent risk factors predicting lower GI bleeding. Because some patients had CKD and progression to ESRD with or without dialysis for a long time, immortal time bias might exist in this condition. Second, our study was based on the ICD-9-CM code. If the doctors did not input a correct or accurate ICD code for CKD, lower GI bleeding, or angiodysplasia bleeding, we might have missed the case or the clinical events. Third, the stage of CKD could not be well classified because no laboratory data were available in the NHIRD database.

In conclusion, dialysis CKD patients have higher risks of lower GI bleeding and angiodysplasia bleeding than the non-CKD subjects and dialysis-free CKD patients. Hemodialysis, dialysis-free CKD, extreme old age, and male gender were independent risk factors for lower GI bleeding. Further prospective well-designed study is warranted.

## Figures and Tables

**Table 1:** Demographic characteristics and comorbidities of CKD-HD, CKD dialysis-free, and control groups.

Variables	Dialysis CKD group (*N* = 574)		Dialysis-free CKD group^∗^(*N* = 1148)		Control^∗^(*N* = 1148)		*P* value
	*n*	%	*n*	%	*n*	%	
*Age (mean* ± *SD*)	61 ± 13.0		62 ± 13.4		61 ± 13.5		0.61
20-44	57	9.9%	93	8.1%	114	9.9%	0.80
45-64	277	48.3%	559	48.7%	554	48.3%	
65-74	146	25.4%	308	26.8%	284	24.7%	
75-84	78	13.6%	163	14.2%	165	14.4%	
≥85	16	2.8%	25	2.2%	31	2.7%	
*Gender*							
Male	318	55.4%	653	56.9%	636	55.4%	0.74
Female	256	44.6%	495	43.1%	512	44.6%	
*Comorbidities*							
Alcoholic liver disease and alcoholism	4	0.7%	6	0.5%	11	1.0%	0.47
Cirrhosis	0	0.0%	0	0.00%	0	0.00%	—
Stroke	57	9.9%	109	9.5%	87	7.6%	0.15
Diabetes mellitus	363	63.2%	739	64.3%	714	62.2%	0.56
Hypertension	363	63.2%	718	62.5%	812	70.7%	<0.001^∗^
Ischemic heart disease	130	22.7%	263	22.9%	276	24.0%	0.75
Congestive heart failure	26	4.5%	55	4.8%	56	4.9%	0.95
Chronic lung disease	91	15.9%	185	16.1%	215	18.7%	0.17
*Charlson score*							
0	140	24.4%	281	24.5%	284	24.7%	<0.001^∗^
1	152	26.5%	404	35.2%	451	39.3%	
2	119	20.7%	218	19.0%	232	20.2%	
≥3	163	28.4%	245	21.3%	181	15.8%	
Charlson score (mean ± SD)	1.7 ± 1.5		1.5 ± 1.4		1.4 ± 1.3		<0.001^∗^
*Medication*							
Aspirin	189	32.9%	389	33.9%	394	34.3%	0.85
Steroids	70	12.2%	143	12.5%	165	14.4%	0.30
Warfarin	7	1.2%	18	1.6%	14	1.2%	0.73
Clopidogrel	11	1.9%	17	1.5%	16	1.4%	0.70
Ticlopidine	7	1.2%	12	1.1%	8	0.7%	0.51

^∗^Match (1 : 2 ratio) with age, sex, comorbidities, and enrollment time. CKD: chronic kidney disease; SD: standard deviation.

**Table 2:** The gastrointestinal bleeding and bleeding-related mortality in dialysis CKD and dialysis-free CKD and control groups during a 6.4-year follow-up period.

Variables	A: dialysis CKD group (*N* = 574)		B: dialysis-free CKD group (*N* = 1148)		C: control group (*N* = 1148)		*P* value		
	*n*	%	*n*	%	*n*	%	A vs. B	A vs. C	B vs. C
All GI bleeding, hospitalized	175	30.5%	126	11.0%	93	8.1%	<0.001^∗^	<0.001^∗^	0.019^∗^
Lower GI bleeding	74	12.9%	41	3.6%	32	2.8%	<0.001^∗^	<0.001^∗^	0.27
Angiodysplasia bleeding	6	1.1%	1	0.1%	1	0.1%	0.003^∗^	0.003^∗^	1.00
Bleeding related mortality	14	2.4%	13	1.13%	0	0	0.0040	<0.001	0.0003

^∗^ *P* < 0.05. CKD: chronic kidney disease; GI: gastrointestinal.

**Table 3:** The independent predictors of lower gastrointestinal bleeding by Cox regression analysis.

Variable	Univariate analysis	95% CI		Multivariate analysis	95% CI	
	Crude HR	Upper	Lower	Adjusted HR	Upper	Lower
Control group	1.00	—	—	1.00	—	—
Dialysis CKD group	5.97	3.83	9.29	29.09	9.66	87.63
Dialysis-free CKD group	1.79	1.07	3.01	6.61	2.27	19.23
Age						
20-44	1.00	—	—	1.00	—	—
45-64	2.91	0.29	29.64	3.00	0.26	34.28
65-74	3.98	0.39	41.14	5.26	0.47	59.08
75-84	3.43	0.34	34.25	5.04	0.44	57.96
≥85	25.59	1.57	416.29	61.47	2.68	1412.10
Male	1.89	1.07	3.33	3.14	1.45	6.78
Stroke	3.13	1.22	8.03	2.41	0.67	8.67
Diabetes mellitus	1.83	0.89	3.79	1.84	0.67	5.05
Hypertension	0.83	0.45	1.50	0.84	0.36	1.98
Ischemic heart disease	0.81	0.46	1.43	0.83	0.38	1.80
Medication						
Aspirin	2.08	1.43	3.01	0.57	0.27	1.20
Steroids	2.03	1.38	2.99	0.75	0.39	1.43
Warfarin	1.50	0.54	4.18	1.28	0.33	5.01
Clopidogrel	2.49	1.51	4.11	1.22	0.58	2.57
Dipyridamole	2.03	1.33	3.10	0.88	0.44	1.75
Ticlopidine	1.23	0.43	3.54	0.40	0.11	1.42
All NSAID	2.00	1.39	2.87	4.00	0.15	107.02
Selective NSAID	1.54	0.91	2.59	0.74	0.35	1.57
Nonselective NSAID	2.01	1.40	2.89	0.11	0.01	2.50

CKD: chronic kidney disease.

## Data Availability

Raw data were generated at the Center for Medical Informatics and Statistics, Kaohsiung Medical University, Kaohsiung, Taiwan. Derived data supporting the findings of this study are available from the corresponding author on request.
